# Compositional Changes in Grapes and Leaves as a Consequence of Smoke Exposure of Vineyards from Multiple Bushfires across a Ripening Season

**DOI:** 10.3390/molecules26113187

**Published:** 2021-05-26

**Authors:** WenWen Jiang, Mango Parker, Yoji Hayasaka, Con Simos, Markus Herderich

**Affiliations:** The Australian Wine Research Institute, Glen Osmond, SA 5064, Australia; maddy.jiang@awri.com.au (W.J.); mango.parker@awri.com.au (M.P.); yoji.hayasaka@awri.com.au (Y.H.); con.simos@awri.com.au (C.S.)

**Keywords:** grape, wine, smoke taint, berry ripening, leaf, glycoside

## Abstract

The negative effects of smoke exposure of grapes in vineyards that are close to harvest are well documented. Volatile phenols in smoke from forest and grass fires can contaminate berries and, upon uptake, are readily converted into a range of glycosylated grape metabolites. These phenolic glycosides and corresponding volatile phenols are extracted into the must and carried through the winemaking process, leading to wines with overtly smoky aromas and flavours. As a result, smoke exposure of grapes can cause significant quality defects in wine, and may render grapes and wine unfit for sale, with substantial negative economic impacts. Until now, however, very little has been known about the impact on grape composition of smoke exposure very early in the season, when grapes are small, hard and green, as occurred with many fires in the 2019–20 Australian grapegrowing season. This research summarises the compositional consequences of cumulative bushfire smoke exposure of grapes and leaves, it establishes detailed profiles of volatile phenols and phenolic glycosides in samples from six commercial Chardonnay and Shiraz blocks throughout berry ripening and examines the observed effects in the context of vineyard location and timing of smoke exposure. In addition, we demonstrate the potential of some phenolic glycosides in leaves to serve as additional biomarkers for smoke exposure of vineyards.

## 1. Introduction

In 2003, an impact on Australian grape and wine quality as a consequence of bushfires was recorded for the first time. Since then, fires in or near viticultural areas have become increasingly frequent [1,2]. Based on the McArthur Forest Fire Danger Index (FFDI), a commonly used measurement of near-surface weather conditions to assess the risks associated with bushfires [3], the daily FFDI value is likely to continue to increase by 15–70% by 2050 in southeast Australia [4,5]. In addition to the increasing frequency of fire events, Australia’s fire season is likely to start earlier in spring (September to November) [6]. Already, more than 60% of Australia recorded its highest daily FFDI values in spring 2019 due to low rainfall, high mean maximum temperature, and many other meteorological and fuel condition factors [7]. The increasing fire danger risk in southeast Australia, which covers most of Australia’s viticulture regions, and the early start of the fire season in spring 2019 together resulted in unprecedented smoke exposure of vineyards. In the Hunter Valley of New South Wales, which produces premium Semillon, Chardonnay, Shiraz and other varietal wines, smoke from multiple bushfires was observed at varying intensities throughout the growing season, from October 2019 until January 2020, and affected grapes at ripening stages from pea-sized green berries to commercial ripeness.

The effects of smoke compounds generated by bushfires, forest fires and prescribed burns on grapevines and the resultant wines have been well studied in the last two decades [1,8,9]. Volatile phenols including phenol, cresol isomers (*ο*-, *m*-, *p*-), guaiacol and syringol are generated by thermal decomposition of lignin sources [1]. They can enter into the grapevine via stomates in the leaves and are absorbed through the berry skin, followed by enzymatic conversion to phenolic glycosides [1,9,10]. Wines made from grapes exposed to smoke contain elevated concentrations of volatile phenols and their glycosides which together cause smoky, ashy, burnt, disinfectant, and medicinal sensory characters for both aroma and palate [8,9,10,11,12]. In addition, the phenolic glycosides can release volatile phenols during winemaking and contribute to smoky flavours in wine [10,13,14,15]. Due to the negative quality impact on a wine’s sensory profile, the smoke-affected grapes may be unsuitable for winemaking, and the rejection of grapes after their exposure to smoke causes significant commercial losses [1,2].

While evaluating the effects of smoke exposure on grapes from Australian vineyards exposed to smoke during the 2009 vintage, Hayasaka and colleagues identified a range of phenolic glycosides in grapes and subsequently established that a number of glycosides, including the abundant syringol and methylsyringol gentiobiosides (GGs, SyGG and MSyGG, respectively), could be used to differentiate between smoke-exposed and non-smoke-exposed vineyards [11,16,17]. Notably, only very low concentrations of phenolic glycosides have been detected in grapes and wine that have not been exposed to smoke [11]. A number of additional monoglucosides (MGs), disaccharides and trisaccharides have been reported in grapes exposed to smoke from fires in the USA, Canada and from model experiments under controlled smoking conditions, but quantitative information about these compounds is lacking [18,19]. The uptake and metabolism of volatile phenols are generally similar between grape varieties, with some differences in volatile phenol glycoside profile between cultivars. For example, Chardonnay grapes were observed to accumulate higher concentrations of phenol pentosylglucosides (PhPGs), cresol pentosylglucosides (CrPGs) and cresol rutinosides (CrRGs) but lower levels of guaiacol gentiobiosides (GuGGs) than Merlot grapes when both varieties were exposed to smoke for the same duration under controlled conditions [13]. In Shiraz berries, Ristic and colleagues [14] found higher levels of guaiacol glycosides, including both MG and disaccharides, compared with Chardonnay berries after smoke exposure. Similarly, smoke-exposed Cabernet Sauvignon and Pinot Noir grapes had lower levels of guaiacol glycosides than Chardonnay and Sauvignon Blanc.

The consequences of smoke exposure at different growing stages post-veraison (after Eichhorn-Lorenz (E-L) stage 35) [20] have been well studied using model smoke experiments [12,21] in ripening grapes that contain an abundance of sugars. In pioneering model experiments and on the basis of guaiacol and 4-methylguaiacol concentrations in the resulting wines, the quality risk from smoke exposure was classified as “low” in vines at the growth stages of 10 cm shoots and flowering, “variable” from pea-sized berries to three days post-veraison, and ‘high’ from seven days post-veraison to harvest [21]. This assessment was made because smoke exposure of pea-sized berries at E-L stage 31 resulted in wines with elevated concentrations of guaiacol and 4-methylguaiacol, and smoky aroma [21,22]. However, these initial experiments on the impacts of early-season exposure were conducted under model conditions and before analytical methods for the measurement of phenolic glycosides in grapes were available. Furthermore, these studies did not include sensory assessment of the smoky off-flavour or aftertaste of the wines on the palate.

From biochemical studies, it is clear that glycosyltransferases play an important role in the metabolism of volatile phenols, and the enzyme UGT72B27 has been shown to convert most smoke-derived phenols to their respective MGs [23]. While glycosyltransferases such as UGT72B27 have been shown to be actively expressed in unripe Gewurztraminer grape berries [17], it has not been determined whether this and other enzymes are active in Shiraz and Chardonnay berries and whether glycosides of smoke-related volatile phenols can be formed under field conditions after smoke exposure of pre-veraison grapes that contain little sugar.

From model experiments using smoke tents it has been established that a positive correlation exists between the intensity and duration of smoke exposure and the degree of smoky aroma and flavour in wine made from grapes exposed to smoke under controlled conditions [15,24,25]. Smoke exposure effects have also been shown to be cumulative: wines were rated similar in smoke sensory characters irrespective of whether they were made from Merlot grapes exposed to low-density smoke for longer periods or high-density smoke for a short period [24]. Another smoke accumulation trial provided further evidence that wine made from grapes exposed to eight repeated smoke events had higher volatile phenols than wine made using grapes exposed to a single smoke event [12]. However, these observations from controlled smoke experiments have not yet been confirmed by analysis of grapes following bushfire smoke events that caused repeated and extensive periods of smoke exposure in vineyards.

Similar to grape berries, grapevine leaves can also take up guaiacol and transform it into glycosides, as proven by stable isotope tracer experiments and also observed after bushfire smoke exposure [10,26]. Glycosyltransferase enzymes have been found in Gewurztraminer leaves which are able to convert guaiacol, 4-methylguaiacol, syringol, 4-methylsyringol, *m*-cresol and *o*-cresol into glycosides [23]; therefore, it is reasonable to assume that grapevine leaves may have a similar response to smoke volatile phenols as grape berries. In contrast to grapes, leaves have a large surface area available for adsorption and take up of volatile phenols from smoke, even very early in the season. However, the suitability of analysis of leaves as source of biomarkers for smoke exposure has not been assessed to date.

This study analysed volatile phenols and their glycosides in commercially grown grapes and leaves exposed throughout 2019 to bushfire smoke from multiple fires in New South Wales in Australia, sampled during the ripening period from E-L stage 27 pre-veraison to E-L stage 38. The impact of smoke on vineyards at different locations and changes in composition during the ripening season are discussed, and the impact on two varieties, Chardonnay and Shiraz, is compared.

## 2. Results and Discussion

### 2.1. Smoke Exposure of Vineyards

The first smoke from the Gospers Mountain Fire in the Wollemi National Park in New South Wales was spotted on 26 October 2019 [27]. In December, the Gospers Mountain Fire merged with multiple fires burning in Yengo National Park, including the Little L fire complex and Corrabare State Forest fire, and with the Kerry Ridge, Paddock Run and the Three Mile Fires. The combined fires affected an estimated one million hectares and during November and December 2019 the Hunter Valley and its vineyards were covered in thick smoke haze [28].

**Figure 1:**
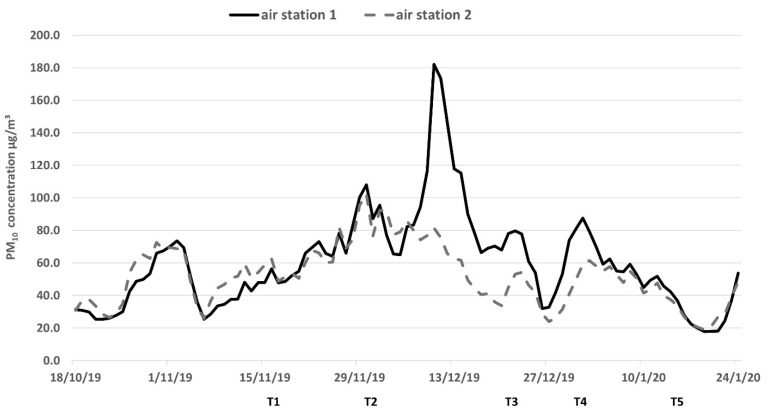
Average particulate matter (PM_10_) concentrations measured by air quality measuring stations near Bulga (air station 1, solid line) and Singleton (air station 2, dashed line) with sampling time points T1 to T5 approximately marked on the time series.

**Figure 2:**
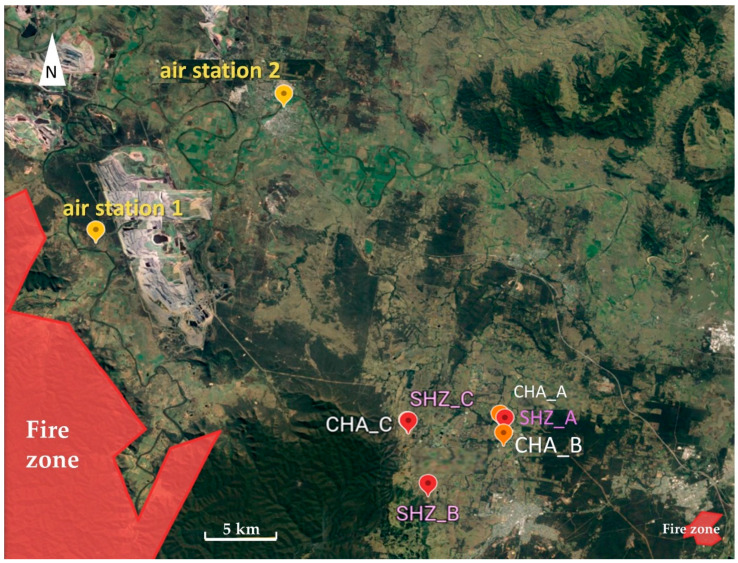
Map [31] of Chardonnay and Shiraz vineyard blocks and air quality monitoring stations (air station 1 near Bulga; air station 2 near Singleton); red areas indicate the closest fire zones. CHA = Chardonnay; SHZ = Shiraz.

The daily average particulate matter (PM_10_) concentrations recorded by public air quality monitoring stations near the Hunter Valley vineyards from 18 October 2019 to 24 January 2020 are plotted in Figure 1 [29]. Both stations are approximately 26 km northwest from the vineyard block C (Figure 2). Overall, the PM_10_ profiles measured by the two stations are similar and indicate continuous and relatively uniform smoke haze across the region, apart from a substantial spike in the middle of December for the data from the air monitoring station 1 near Bulga. Prior to the fires commencing, the PM_10_ levels from 18 to 25 October 2019 were below 50 µg/m^3^, which indicates low air pollution [30]. There was an increase in the daily average PM_10_ concentrations to 70 µg/m^3^ between 26 October and 2 November 2019 at both stations; the early smoke haze likely reflected the impact of the Gospers Mountain fire burning ~70 km to the west of the vineyards, which started on 26 October 2019 and continued to burn for 79 days. The smoke haze and PM_10_ data then steadily increased to approximately 100 µg/m^3^ by the end of November. In early December, the average daily PM_10_ concentration increased to 182 µg/m^3^ measured by station 1 at Bulga on 10 December, which was almost double the data acquired by station 2 near Singleton, suggesting that the smoke was denser in western locations. The differences in air quality between the two stations were most likely a consequence of wind direction and location relative to fire activity. Western station 1 recorded a predominantly south-easterly wind direction which would have blown smoke from the nearby Corrabare State Forest fires towards the vineyard blocks, whereas station 2 recorded a north-easterly wind direction, which is not closely associated with fire activity [29]. Heavy haze and a strong smoky smell were observed in the vineyards from 6 to 16 December; after that date, the PM_10_ levels dropped roughly 10 µg/m^3^ per day. In summary, the period of the most severe smoke exposure started when the vines had pea-sized green berries and continued until veraison. From the end of December 2019, the PM_10_ concentration dropped slowly below 50 µg/m^3^, indicating that the grapevines were exposed to minimal or no smoke from veraison until harvest. During January 2020, for most days no haze or smoky smells were recorded in the vineyards.

### 2.2. Smoke Exposure Markers in Grapes

In order to assess the impact of smoke exposure on unripe grapes very early in the growing season, we quantified free volatile phenols and their known glycosidic grape metabolites in samples collected throughout ripening from the two main varieties: Chardonnay and Shiraz (Table 1). The location of the blocks had been chosen to assess variety and site effects. In addition to grapes, leaves were also collected and analysed to verify smoke exposure at a vineyard level and complement the PM_10_ (haze) data. The comparison of smoke exposure markers between leaves and grapes was also thought to assist with differentiating between the potential effects of grape maturity and site-specific smoke exposure. Volatile phenols commonly found in smoke from grass and forest fires [1], namely, guaiacol; 4-methylguaiacol; syringol; methylsyringol; and *o*-, *m*- and *p*-cresol, were analysed in grapes sampled at T1 to T4, and in leaves at T1, and were below the limit of quantitation (LoQ) of 1 µg/kg, except 2 µg/kg for syringol and methylsyringol in all samples measured (data not shown). This is not surprising given that volatile phenol concentrations have been demonstrated to decrease rapidly in grapes following smoke exposure [15]; it confirms that absence of elevated volatile phenols in grapes cannot rule out smoke exposure of grapes and smoke taint in wine [32]. In contrast to the free volatile phenols, a broad range of their glycosidic grape metabolites as described earlier [10,11] could be detected in grapes from all five sampling time points and leaves sampled at three time points (T1, T3 and T5). GGs of phenol and cresol, and MGs of methylguaiacol and methylsyringol, were below or at the LoQ (1 µg/kg) in all grapes and leaves analysed and were excluded from the subsequent data analysis.

**Figure 3:**
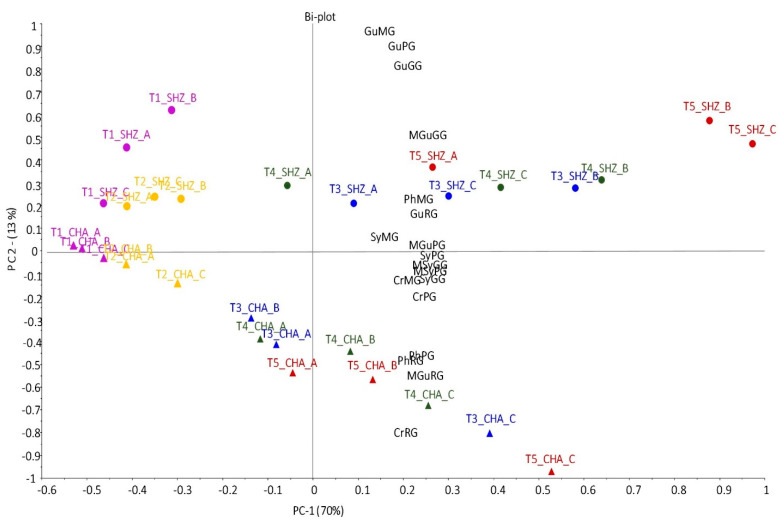
Principal components PC-1 and PC-2 scores and loadings biplot of phenolic glycosides in Chardonnay (CHA; triangles) and Shiraz (SHZ; circles) grape berries from all blocks sampled at five time points (T1: purple; T2: yellow; T3: blue; T4: green; T5: red). All values (ng/berry) are means of three field replicates (*n* = 3) and expressed as syringol gentiobioside equivalents per berry. Gu = guaiacol; Cr = cresol; Ph = phenol; Sy = syringol; MGu = 4-methylguaiacol; MSy = 4-methylsyringol; MG = monoglucosides; GG = gentiobiosides; PG = pentosylglucosides; RG = rutinoside.

During berry development and ripening, the size of grape berries increases (Table 2). In addition, berry size also reflects differences between varieties and vineyard management practices. Therefore, principal component analysis (PCA) was performed using the average grape phenolic glycoside content expressed as ng per berry. This enabled comparisons between berries of differing size, from both varieties, grown on six blocks and collected across all five time points. Figure 3 shows the biplot of the first two principal components (PCs) which account for a total of 83% explained variance. PC-1 explains 70% of the total variance in the phenolic glycoside profiles and indicates the discrimination of the grape berry samples by time point. Grapes from both varieties sampled at T1 and T2 across the six blocks all have low levels of phenolic glycosides and are grouped together on the left side of the PCA plot. In contrast, the content of phenolic glycosides per berry was high in grapes sampled from T3 to T5 from all blocks, with the highest samples positioned on the right-hand side of the PCA plot. The cumulative effect of smoke exposure throughout ripening explains the highest glycosides content (ng/berry) found in T5 grape samples and confirms the results reported previously for model smoke experiments with controlled degrees of exposure [12]. The increase over time in glycosides per berry during ripening reflects the intensity of smoke in the region, closely following the pattern of PM_10_ data shown in Figure 1. While elevated, yet relatively low, levels of smoke haze from the distant Gospers Mountain fire were observed before T2, it is the heavy smoke (Figure 1) experienced from T3 to T5 (Table 1) which is associated with the substantial increase in phenolic glycoside levels per berry after T3, reaching maxima of 73 ng/berry and 87 ng/berry of SyGG in Chardonnay and Shiraz grapes, respectively, from block C sampled at T5 (Table S1, in the Supplementary Materials). The maximum grape SyGG concentrations in µg per kg (Table S2) observed in this study were similar to previous results which found the SyGG ranging from 68 to 1623 µg/kg in grapes exposed to smoke from bushfires in Victoria during 2009 [11].

Differences due to vineyard location and variety became more apparent later in the season. Among the samples from T3 to T5, both Chardonnay and Shiraz grapes from block A were always lower in phenolic glycosides than samples from blocks B and C at the same time point. Similarly, co-located Chardonnay and Shiraz samples from block C were the highest in phenolic glycosides per berry at T5 (Table S1). The only exception from ranking the smoke exposure of vineyards according to their phenolic glycosides in the order of block A < block B < block C was observed for Shiraz grown on block B, which exceeded Shiraz C at T3 and T4. In summary, the observed differences in glycoside levels between vineyards were largely independent of variety and likely reflected site effects caused by differences in smoke exposure (Table S3, using the analysis of variance of SyGG per berry basis as an example), as indicated by the differences between the air quality data during December (Figure 1).

The PCA in Figure 3 demonstrates smaller differences between the varieties compared to ripening and exposure time effects as illustrated by the spread along PC-2, which explains 13% of variance, with some notable differences between Chardonnay and Shiraz increasing from T3 to T5 along with berry maturity. In samples from co-located blocks, Chardonnay grapes were higher in RGs of phenol, cresols and 4-methylguaiacol and PhPGs after smoke exposure. On the other hand, guaiacol MGs, GGs and PGs were most abundant in Shiraz grapes sampled after T3, which is in agreement with previous findings [11]. Factors such as differences in skin and cuticular waxes; berry physiology [33]; distribution of glycosides between skin, juice and pulp [10]; and the variation in the expression and activity of glycosyltransferase enzymes [23] could explain the differences between Chardonnay and Shiraz grapes observed in this study and would warrant further investigations into uptake and metabolism of smoke-derived phenols by different cultivars.

In the present study, SyGG and MSyGG (together with a number of the minor phenolic glycosides) were located close to the centre of the PCA plot in Figure 3 and not separated by PC-2, suggesting that concentrations of SyGG and MSyGG are largely independent of variety effects. This further supports their important role as smoke exposure markers in grapes [11,15,16,33].

To assess potential variations in the glycosylation reaction throughout ripening, a correlation matrix was calculated for each variety and the phenolic glycosides detected in grapes (expressed as ng/berry, Table S4). For Chardonnay, all the glycosides, apart from MGs, were highly co-correlated with a correlation coefficient greater than or close to 0.7. Similarly for Shiraz, MGs content in grapes was poorly correlated with most disaccharides. However, RGs in Shiraz grapes were well correlated with GGs but weakly correlated with PGs, apart from PhPGs, as observed previously [11]. The weak correlation between MGs and disaccharides could indicate that MGs do not represent biosynthetic endpoints and do not accumulate like disaccharides, but rather are intermediates which are further glycosylated or are subjected to other metabolic reactions. Overall, the differences in correlations between Chardonnay and Shiraz align with the compositional differentiation between varieties observed in the PCA plot (Figure 3). In summary, the correlations between individual phenolic glycosides observed in this study are in line with published results of grapes exposed to bushfire smoke close to harvest and indicate that the uptake of volatile phenols and glycosylation response of a grape berry are relatively consistent across the ripening season, in particular for SyGG, MSyGG and the RGs [11].

### 2.3. Smoke Exposure Markers in Leaves

Grapevine leaves have a large surface area even early in the ripening season, are able to adsorb volatile phenols and have been proven to form guaiacol glycosides in model experiments [10,26]. In addition, glycosyltransferase enzymes have been found in Gewurztraminer leaves which are able to metabolize a range of volatile phenols [23]. Therefore, we hypothesized that grapevine leaves might be suitable for assessing smoke exposure of vines and could provide additional information that might aid interpretation of berry ripening effects.

**Figure 4:**
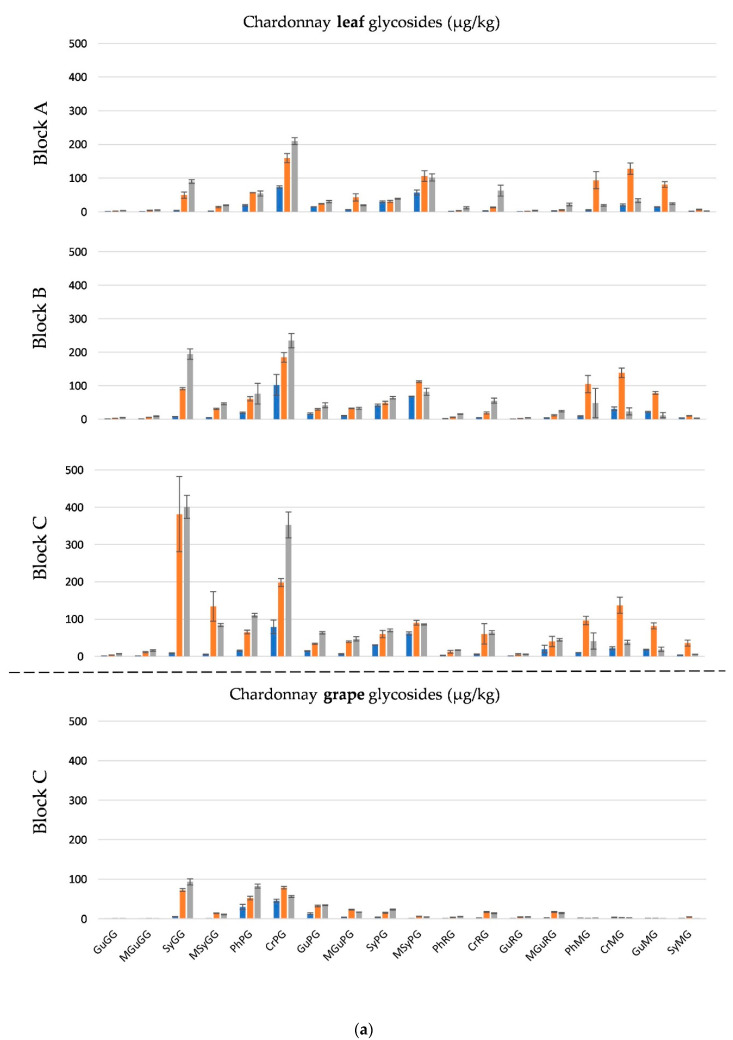

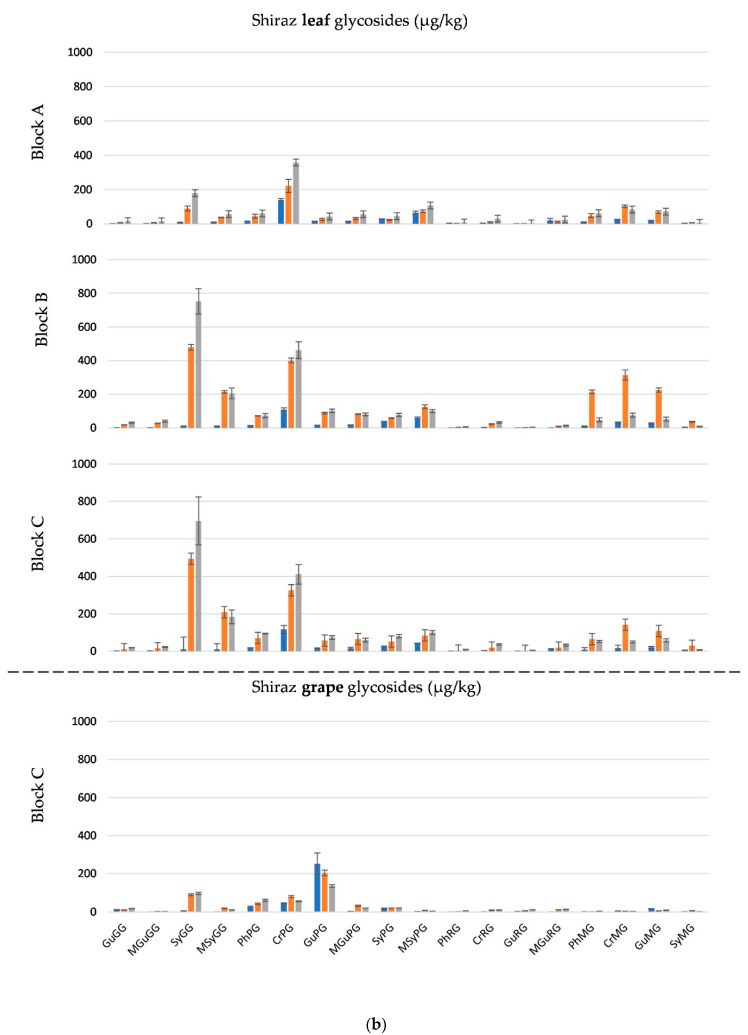
(**a**) Concentrations of phenolic glycosides in Chardonnay leaves from all sites and in grapes from block C sampled at time points T1 (blue bars), T3 (orange bars) and T5 (grey bars). Values are means of three vineyard replicates (*n* = 3) expressed in µg/kg as syringol gentiobioside equivalents. Error bars denote ± standard error. Gu = guaiacol; Cr = cresol; Ph = phenol; Sy = syringol; MGu = 4-methylguaiacol; MSy = 4-methylsyringol; MG = monoglucosides; GG = gentiobiosides; PG = pentosylglucosides; RG = rutinoside. (**b**) Concentrations of phenolic glycosides in Shiraz leaves from all sites and in grapes from block C sampled at time points T1 (blue bars), T3 (orange bars) and T5 (grey bars). Values are means of three vineyard replicates (*n* = 3) expressed in µg/kg as syringol gentiobioside equivalents. Error bars denote ± standard error. Gu = guaiacol; Cr = cresol; Ph = phenol; Sy = syringol; MGu = 4-methylguaiacol; MSy = 4-methylsyringol; MG = monoglucosides; GG = gentiobiosides; PG = pentosylglucosides; RG = rutinoside.

The concentration of phenolic glycosides in Chardonnay and Shiraz leaves from all blocks and Chardonnay and Shiraz berries from block C sampled at timepoints T1, T3 and T5 are shown in Figure 4a,b, respectively, and are expressed on a per kg basis to allow comparison between berries and leaves. Generally, the glycoside levels were low in leaves from all the T1 samples; this aligned well with grape glycoside results for T1 and was indicative of the low level of smoke exposure at this time as the PM_10_ concentrations measured before 15 November 2019 were all lower than 100 µg/m^3^ (Figure 1). However, in parallel with the increase of PM_10_ levels in early December in 2019, the glycosides levels in leaf samples from both varieties, particularly SyGG and MSyGG, increased sharply at T3 followed by a smaller increase from T3 to T5 (Table S5). Notable exceptions include CrPGs, which were highly abundant in leaves at T1, and in berries, but did not increase over time as observed for the other glycosides, as well as guaiacol pentosylglucosides, which were present at high concentrations in Shiraz berries at T1 and decreased in concentration over time. This might point towards CrPGs being “housekeeping” glycosides in leaves and berries which do not reflect environmental smoke exposure.

For co-located Chardonnay and Shiraz samples from blocks A and C, the glycoside concentrations in the leaf samples were much higher than those in the grape berry samples, in agreement with results reported previously [26]. This was likely due to the larger surface area of the leaves compared to the berries [34]. Furthermore, the relative expression of glucosyltransferase has shown to be higher in leaves than in grape berries [23,35]. MGs in leaves increased at T3 after exposure to heavy smoke, but were lower at T5. The decrease of MGs in leaves towards ripening could be due to subsequent glycosylation, forming disaccharides, or other sequestration reactions.

Comparing the two varieties, after smoke exposure, Shiraz leaves from blocks A and C had higher glycoside concentrations than Chardonnay leaves grown nearby (Table S5), which is consistent with the varietal response observed for phenolic glycosides in the berries. Notably, Shiraz and Chardonnay in block B were not co-located, but approximately 7 km apart, with Shiraz block B near Shiraz block C in the western and more heavily smoke-exposed part of the region, while Chardonnay block B was located near Chardonnay block A in the east (Figure 2). In relation to a possible role of leaves as a sentinel for monitoring environmental smoke exposure of vines, it was noted that the profile and concentration of phenolic glycosides in Shiraz leaves from block B resembled data from nearby Shiraz block C and phenolic glycosides in Chardonnay leaves from block B resembled data from nearby Chardonnay block A. While essential knowledge of glycoside concentration in leaves not exposed to smoke is currently lacking, these observations demonstrate the potential of some phenolic glycosides in leaves to serve as additional biomarkers for smoke exposure of vineyards and suggest that analysis of phenolic glycosides in leaves might be suitable for differentiating smoke exposure at a vineyard level.

## 3. Materials and Methods

### 3.1. Grape Berry and Leaf Sampling

Six commercial vineyard blocks in the Hunter Valley in New South Wales, Australia, which had been first exposed to smoke from the Gospers Mountain Fire in late October 2019, were selected for this study (Figure 2). Each block is planted with Chardonnay or Shiraz; in vineyards A and C, the Shiraz and Chardonnay blocks are adjacent to each other. The Chardonnay block B (CHA_B) is relatively closely located to vineyard A with CHA_A and SHZ_A, but Shiraz block B (SHZ_B) is ~6 km to the south-west of vineyard C with CHA_C and SHZ_C. Smoke haze data were retrieved from New South Wales (NSW) government air quality measuring stations at Bulga (air station 1) and Singleton (air station 2) ~26 km north-west of the vineyards [29]. As a proxy for smoke exposure in the region, particulate matter measurements (PM_10_) averaged over 24 h and expressed in µg/m^3^ were used and graphed as a moving average over five days. Fortnightly, sampling of grapes and leaves commenced approximately three weeks after the first observations of smoke exposure (T1 to T3) and continued post-veraison (T4) to commercial maturity (T5) (Table 1). At each time point (Table 1), three replicate samples per block, each of five grape bunches and 15 whole leaves with similar size, were randomly collected from different panels. Grape sampling commenced when berries were approximately 2 mm in diameter and continued to commercial maturity. All grape bunches and leaves were immediately frozen by the industry partners, transported frozen and stored at −20 °C until the day of sample preparation.

### 3.2. Sample Preparation

Each grape berry sample was destemmed by hand and between 100 and 500 berries were counted, depending on berry size and E-L stage, then weighed on the balance to determine the average berry mass before homogenization (T18 Ultra Turrax, IKA, Staufen, Germany). Three independently replicated vineyard berry samples were prepared for each block and time point. Grape berry homogenate extraction for analysis of phenolic glycosides by liquid chromatography-tandem mass spectrometry (HPLC-MS/MS) was performed according to the stable isotope dilution analysis (SIDA) protocol published previously [11]. Leaf samples from T1, T3 and T5 for each block were ground under liquid nitrogen using an IKA A11 basic analytical mill (IKA^®^ Works, Inc., NC). Three replicate leaf samples were prepared for each block and time point with a method similar to that described previously [10]. Briefly, 1 g of leaf powder was weighed into a 10 mL plastic tube (Sarstedt Australia Pty Ltd., SA, Australia) then spiked with d_3_-syringyl-β-D-gentiobioside as internal standard followed by adding 5 mL of MilliQ water. Vials were mixed for 15 min using a rotary tube mixer (Ratek Instruments, VIC, Australia), centrifuged at 3750 rpm for 10 min (Allegra^®^ X-12R centrifuge, Beckman Coulter, CA, USA), then the supernatant was loaded onto pre-conditioned Extra Clean C18-HF SPE 500 mg/4 mL cartridges (Sstarpure, Singapore). Another 5 mL of MilliQ water was added to the pellet remaining in the tube; this was re-extracted a second time using the same procedure as above, and the re-extracted supernatant was loaded onto the same SPE cartridge. The solid phase extraction and the reconstitution followed the steps described by Hayasaka and colleagues [10].

### 3.3. Chemical Analysis

Analytical-grade chemicals were purchased from Sigma Aldrich (Steinheim, Germany and Castle Hill, NSW, Australia) and solvents (HPLC grade) were purchased from Merck (Victoria, Australia). Grape and leaf sample extracts were analysed for phenolic glycosides by an Agilent 1200 HPLC system (Agilent Technologies, Forest Hill, Vic., Australia) equipped with 1290 binary pump combined with an AB SCIEX Triple Quad^TM^ 4500 tandem mass spectrometer with a Turbo V^TM^ ion source (Framingham, MA, USA). The instrument operating conditions and method validation were reported previously [11]. Data acquisition and processing were performed using Analyst software (version 1.7.3 AB SCIEX). Volatile phenols (guaiacol, 4-methylguaiacol, *o*-, *m*- and *p*-cresol, syringol and 4-methylsyringol) were analysed by the Australian Wine Research Institute’s (AWRI’s) Commercial Services Laboratory (Adelaide, SA, Australia) as reported previously [36] using an Agilent 6890 gas chromatograph coupled to a 5973 mass selective detector. The LoQ for volatile phenols was 1–2 µg/kg depending on the analyte, whilst the LoQ for phenolic glycosides was 1 µg/kg.

### 3.4. Data Analysis

The mean and standard error of phenolic glycoside concentrations in grape berries and leaves from each block and each time points were calculated with GraphPad Prism (version 9, GraphPad Software, CA, USA). Principal component analysis (PCA) was performed with The Unscrambler (version 11, CAMO Process AS, Trondheim, Norway) using the concentration (ng/berry) of phenolic glycosides in grape berries. The glycosides correlation matrix and one way analysis of variance were established in MiniTab (version 20, Minitab Ltd., Coventry, UK) using linear regression models. Mean comparisons were performed by Tukey test at *p* < 0.05.

## 4. Conclusions

This study determined the consequences for grape composition of smoke exposure of vineyards from multiple wildfires which commenced at flowering, continued through fruit set (2 mm berries, E-L stage 27) and beyond veraison (E-L stage 35). The data unequivocally established that a range of phenolic glycosides can be formed as metabolites in unripe grapes and also in leaves of *Vitis* *vinifera* as a consequence of exposure to smoke.

In very small grape berries sampled prior to E-L stage 33, only very low concentrations of phenolic glycosides were detected, despite anecdotal evidence and PM_10_ data demonstrating vineyard exposure to some smoke haze. As the phenolic smoke exposure markers were absent from leaves sampled at the same time, the results for the grapes from the very early ripening samples suggest a level of volatile taint compounds in smoke that was too low to cause an obvious contamination. Alternatively, the unripe small grapes might have been impaired in their biochemical ability to take up the volatile phenols or they could have formed other yet to be identified metabolites.

In both Chardonnay and Shiraz, unripe green grapes sampled at E-L stage 33 prior to veraison and throughout ripening, as well as in leaves, had clearly elevated concentrations of a range of phenolic glycosides and profiles of known smoke exposure markers SyGG, MSyGG and RGs reflected the smoke haze observed in the region, irrespective of variety.

Notably, the consequences of cumulative smoke exposure from multiple wildfires and the unknown relationship between smoke haze (i.e., particles), and presence of taint compounds among the volatile organic compounds in smoke, make it impossible to accurately relate the appearance of the phenolic smoke exposure markers in grapes to the timing of a specific smoke event. It is also not possible to predict from the available data at what early phenology stages smoke exposure represents no risk or only a very low risk of contaminating grapes and causing quality defects in the resultant wine.

In summary, the influence of early smoke exposure on grapes and vines, and also on wine sensory attributes, warrants further investigation. Still, this study has shown that uptake of volatile phenols from smoke and accumulation of the phenolic glycosides in green unripe grape berries may occur, with several key glycosides detected in green berries at concentrations similar to the concentrations found in smoke-exposed ripe grapes. The results represent an important step towards an improved understanding of the changes in volatile phenols and their glycosides in grapes after early-season smoke exposure, as opposed to waiting until grapes are mature before estimating smoke impacts, and will help growers to make better informed vineyard management decisions that could help save costs from growing grapes which are unfit for sale and winemaking.

## Figures and Tables

**Table 1:** Dates for grape berry and leaf sampling and the ripening E-L stage of each time point.

Time Point	Sampling Date	E-L Stage					
		CHA_A	CHA_B	CHA_C	SHZ_A	SHZ_B	SHZ_C
T1	15/11/2019	30–31	27–32	31	27–29	27–29	27–29
T2	29/11/2019	32	32	32	32	31	32
T3	16/12/2019	33	33	33	33	33	33
T4	28/12/2019	35	35	35	35	35	35
T5	10/01/2020	38	38	38	37–38	37–38	37–38

CHA = Chardonnay; SHZ = Shiraz.

**Table 2:** Average berry mass (g) of Chardonnay and Shiraz grapes sampled from T1 to T5.

Variety	Block	Time Point				
		T1	T2	T3	T4	T5
Chardonnay	A	0.23	0.42	0.51	0.84	1.03
	B	0.20	0.35	0.35	0.64	0.73
	C	0.30	0.40	0.66	0.78	0.79
Shiraz	A	0.12	0.40	0.69	0.82	1.14
	B	0.16	0.32	0.48	0.64	0.82
	C	0.17	0.32	0.45	0.72	0.80

Value are means of three replicates (*n* = 3) with each sample comprising 100–500 berries depending on the growing stage.

## Data Availability

The data presented in this study are available in supplementary material.

## Supplementary Materials

The following are available online. Table S1: Phenolic glycosides (ng/berry) in Chardonnay and Shiraz grapes sampled from T1 to T5. Table S2: Concentrations of phenolic glycosides (µg/kg) in Chardonnay and Shiraz grapes sampled from T1 to T5. Table S3: Analysis of variance summary of syringol gentiobioside (ng/berry) in grape berries across the data set at 95% confidence. Table S4: Correlation matrix of phenolic glycosides concentrations in ng per berry from grapes of Chardonnay (a) and Shiraz (b). Table S5: Analysis of variance summary of syringol gentiobioside (µg/kg) in Chardonnay and Shiraz leaves sampled from different blocks in three time points at 95% confidence.

## Sample Availability

Samples of the compounds are not available from the authors.
