# Supplementary material for: Compositional Changes in Grapes and Leaves as a Consequence of Smoke Exposure of Vineyards from Multiple Bushfires across a Ripening Season
Source: Molecules. 2021 May 26;26(11):3187. doi: 10.3390/molecules26113187 (PMC8197810; doi:10.3390/molecules26113187)
Supplement: Supplementary file 1 [file molecules-26-03187-s001.zip › molecules-1204092-supplementary.pdf]

Supplementary Materials

Compositional Changes in Grapes and Leaves as a Consequence of Smoke Exposure of Vineyards from Multiple Bushfires across a Ripening Season

WenWen Jiang, Mango Parker, Yoji Hayasaka, Con Simos and Markus Herderich*

The Australian Wine Research Institute, SA 5064, Glen Osmond, Australia; maddy.jiang@awri.com.au (W.J.); mango.parker@awri.com.au (M.P.); yoji.hayasaka@awri.com.au (Y.H); con.simos@awri.com.au (C.S.)
* Correspondence: markus.herderich@awri.com.au; Tel: +61-8-8313-6600

Table 1. Phenolic glycosides (ng / berry) in Chardonnay and Shiraz grapes sampled from T1 to T5.

Variety	Time Point	Vineyard	PhGG	CrGG	GuGG	MGuGG	SyGG	MSyGG	PhPG	CrPG	GuPG	MGuPG	SyPG	MSyPG	PhRG	CrRG	GuRG	MGuRG	PhMG	CrMG	GuMG	MGuMG	SyMG	MSyMG
Chardonnay	T1	A	<LoQ	<LoQ	<LoQ	<LoQ	<LoQ	<LoQ	2	2	<LoQ	<LoQ	<LoQ	<LoQ	<LoQ	<LoQ	<LoQ	<LoQ	<LoQ	<LoQ	<LoQ	<LoQ	<LoQ	<LoQ
		B	<LoQ	<LoQ	<LoQ	<LoQ	<LoQ	<LoQ	2	4	<LoQ	<LoQ	<LoQ	<LoQ	<LoQ	<LoQ	<LoQ	<LoQ	<LoQ	<LoQ	<LoQ	<LoQ	<LoQ	<LoQ
		C	<LoQ	<LoQ	<LoQ	<LoQ	1	<LoQ	7	12	4	<LoQ	1	<LoQ	<LoQ	<LoQ	<LoQ	<LoQ	<LoQ	<LoQ	<LoQ	<LoQ	<LoQ	<LoQ
	T2	A	<LoQ	<LoQ	<LoQ	<LoQ	<LoQ	<LoQ	6	14	4	1	1	<LoQ	<LoQ	<LoQ	<LoQ	<LoQ	<LoQ	1	<LoQ	<LoQ	<LoQ	<LoQ
		B	<LoQ	<LoQ	<LoQ	<LoQ	<LoQ	<LoQ	8	14	3	1	1	<LoQ	<LoQ	<LoQ	<LoQ	<LoQ	<LoQ	<LoQ	<LoQ	<LoQ	<LoQ	<LoQ
		C	<LoQ	<LoQ	<LoQ	<LoQ	4	<LoQ	10	20	7	3	2	<LoQ	<LoQ	2	<LoQ	2	<LoQ	1	<LoQ	<LoQ	<LoQ	<LoQ
	T3	A	<LoQ	<LoQ	<LoQ	<LoQ	13	2	18	23	11	8	4	2	1	6	2	5	<LoQ	2	<LoQ	<LoQ	1	<LoQ
		B	<LoQ	<LoQ	<LoQ	<LoQ	18	2	13	24	10	6	4	1	<LoQ	5	1	4	<LoQ	2	<LoQ	<LoQ	1	<LoQ
		C	<LoQ	<LoQ	<LoQ	<LoQ	47	9	34	51	21	15	10	4	2	11	3	11	<LoQ	2	<LoQ	<LoQ	3	<LoQ
	T4	A	<LoQ	<LoQ	<LoQ	<LoQ	15	2	26	22	11	6	5	1	2	5	2	4	<LoQ	1	<LoQ	<LoQ	<LoQ	<LoQ
		B	<LoQ	<LoQ	<LoQ	<LoQ	26	3	30	33	15	9	6	2	1	5	2	5	1	3	<LoQ	<LoQ	<LoQ	<LoQ
		C	<LoQ	<LoQ	<LoQ	<LoQ	56	8	31	29	18	11	14	3	2	8	3	10	<LoQ	2	<LoQ	<LoQ	<LoQ	<LoQ
	T5	A	<LoQ	<LoQ	<LoQ	<LoQ	21	3	40	27	16	7	8	2	3	6	2	5	<LoQ	<LoQ	<LoQ	<LoQ	<LoQ	<LoQ
		B	<LoQ	<LoQ	<LoQ	<LoQ	36	4	46	37	19	9	9	2	2	6	2	5	<LoQ	2	<LoQ	<LoQ	<LoQ	<LoQ
		C	<LoQ	<LoQ	<LoQ	<LoQ	73	8	65	44	27	13	18	4	4	10	4	11	1	2	<LoQ	<LoQ	<LoQ	<LoQ
Shiraz	T1	A	<LoQ	<LoQ	2	<LoQ	<LoQ	<LoQ	3	6	35	<LoQ	2	<LoQ	<LoQ	<LoQ	<LoQ	<LoQ	<LoQ	<LoQ	5	<LoQ	<LoQ	<LoQ
		B	<LoQ	<LoQ	2	<LoQ	1	<LoQ	7	12	65	<LoQ	4	<LoQ	<LoQ	<LoQ	<LoQ	<LoQ	<LoQ	1	7	<LoQ	<LoQ	<LoQ
		C	<LoQ	<LoQ	1	<LoQ	<LoQ	<LoQ	3	5	24	<LoQ	2	<LoQ	<LoQ	<LoQ	<LoQ	<LoQ	<LoQ	<LoQ	2	<LoQ	<LoQ	<LoQ
	T2	A	<LoQ	<LoQ	1	<LoQ	<LoQ	<LoQ	6	9	34	<LoQ	3	<LoQ	<LoQ	<LoQ	<LoQ	<LoQ	<LoQ	<LoQ	1	<LoQ	<LoQ	<LoQ
		B	<LoQ	<LoQ	1	<LoQ	2	<LoQ	8	19	55	2	6	<LoQ	<LoQ	<LoQ	<LoQ	<LoQ	<LoQ	<LoQ	1	<LoQ	<LoQ	<LoQ
		C	<LoQ	<LoQ	2	<LoQ	1	<LoQ	6	14	53	2	5	<LoQ	<LoQ	<LoQ	<LoQ	<LoQ	<LoQ	<LoQ	1	<LoQ	<LoQ	<LoQ
	T3	A	<LoQ	<LoQ	3	<LoQ	13	3	24	45	80	11	10	3	<LoQ	2	1	2	<LoQ	2	2	<LoQ	2	<LoQ
		B	<LoQ	<LoQ	5	1	52	12	29	56	115	24	13	5	<LoQ	4	2	5	<LoQ	2	3	<LoQ	4	<LoQ
		C	<LoQ	<LoQ	5	<LoQ	40	9	19	37	91	15	9	4	<LoQ	4	3	5	<LoQ	2	2	<LoQ	3	<LoQ
	T4	A	<LoQ	<LoQ	3	<LoQ	14	2	20	23	63	7	7	2	<LoQ	1	1	2	<LoQ	2	4	<LoQ	<LoQ	<LoQ
		B	<LoQ	<LoQ	7	1	71	10	38	49	99	20	17	4	1	4	3	6	2	3	5	<LoQ	2	<LoQ
		C	<LoQ	<LoQ	6	<LoQ	53	9	27	35	80	16	13	4	<LoQ	4	3	6	1	3	4	<LoQ	1	<LoQ
	T5	A	<LoQ	<LoQ	7	<LoQ	25	3	30	29	76	9	10	2	2	4	4	5	1	2	6	<LoQ	<LoQ	<LoQ
		B	<LoQ	<LoQ	12	2	87	11	45	45	114	20	19	4	3	6	7	8	2	2	7	<LoQ	1	<LoQ
		C	<LoQ	<LoQ	14	2	77	9	49	44	108	17	17	4	5	8	10	11	3	2	7	<LoQ	1	<LoQ

All values (ng/berry) are means of three replicates (*n* = 3) and expressed as syringol gentiobioside equivalents per berry. Gu = guaiacol; Cr = cresol; Ph = phenol; Sy = syringol; MGu = 4-methylguaiacol; MSy = 4-methylsyringol; MG = monoglucosides; GG = gentiobiosides; PG = pentosylglucosides; RG = rutinoside. LoQ = limit of quantitation (1 μg/kg).

Table 2. Concentrations of phenolic glycosides (µg/kg) in Chardonnay and Shiraz grapes sampled from T1 to T5.

Variety	Time Point	Vineyard	PhGG	CrGG	GuGG	MGuGG	SyGG	MSyGG	PhPG	CrPG	GuPG	MGuPG	SyPG	MSyPG	PhRG	CrRG	GuRG	MGuRG	PhMG	CrMG	GuMG	MGuMG	SyMG	MSyMG
Chardonnay	T1	A	<LoQ	<LoQ	<LoQ	<LoQ	3	<LoQ	28	37	11	3	4	<LoQ	<LoQ	1	<LoQ	<LoQ	2	6	1	<LoQ	<LoQ	<LoQ
		B	<LoQ	<LoQ	<LoQ	<LoQ	4	<LoQ	19	34	8	3	3	<LoQ	<LoQ	1	<LoQ	<LoQ	<LoQ	6	1	<LoQ	1	<LoQ
		C	<LoQ	<LoQ	<LoQ	<LoQ	5	<LoQ	29	45	13	4	4	<LoQ	<LoQ	2	<LoQ	2	1	3	<LoQ	<LoQ	<LoQ	<LoQ
	T2	A	<LoQ	<LoQ	<LoQ	<LoQ	2	<LoQ	15	33	9	3	3	<LoQ	<LoQ	1	<LoQ	<LoQ	<LoQ	3	<LoQ	<LoQ	2	<LoQ
		B	<LoQ	<LoQ	<LoQ	<LoQ	3	<LoQ	23	41	9	3	3	<LoQ	<LoQ	1	<LoQ	<LoQ	2	2	<LoQ	<LoQ	<LoQ	<LoQ
		C	<LoQ	<LoQ	<LoQ	<LoQ	10	1	27	51	18	9	6	2	1	4	1	4	1	3	<LoQ	<LoQ	2	<LoQ
	T3	A	<LoQ	<LoQ	<LoQ	<LoQ	27	4	35	45	21	15	8	3	2	12	3	9	<LoQ	5	<LoQ	<LoQ	3	<LoQ
		B	<LoQ	<LoQ	<LoQ	<LoQ	52	7	38	69	27	17	12	4	3	13	4	12	2	4	1	<LoQ	4	<LoQ
		C	<LoQ	<LoQ	<LoQ	<LoQ	72	13	52	78	32	22	15	6	3	17	4	17	<LoQ	3	1	<LoQ	5	<LoQ
	T4	A	<LoQ	<LoQ	<LoQ	<LoQ	19	2	31	27	13	7	6	2	2	6	2	5	<LoQ	2	<LoQ	<LoQ	<LoQ	<LoQ
		B	<LoQ	<LoQ	<LoQ	<LoQ	40	5	47	51	23	14	10	3	2	8	3	9	2	5	1	<LoQ	1	<LoQ
		C	<LoQ	<LoQ	<LoQ	<LoQ	72	10	40	38	23	13	17	4	3	11	3	12	<LoQ	2	<LoQ	<LoQ	1	<LoQ
	T5	A	<LoQ	<LoQ	<LoQ	<LoQ	21	3	39	27	15	6	8	2	3	6	2	5	<LoQ	<LoQ	<LoQ	<LoQ	<LoQ	<LoQ
		B	<LoQ	<LoQ	<LoQ	<LoQ	48	5	63	51	27	12	13	3	3	8	3	7	1	2	<LoQ	<LoQ	<LoQ	<LoQ
		C	<LoQ	<LoQ	<LoQ	<LoQ	93	11	82	56	34	16	23	4	6	13	5	14	2	2	<LoQ	<LoQ	<LoQ	<LoQ
Shiraz	T1	A	<LoQ	<LoQ	16	<LoQ	5	<LoQ	26	52	292	4	21	2	<LoQ	<LoQ	2	<LoQ	<LoQ	5	44	<LoQ	1	<LoQ
		B	<LoQ	<LoQ	11	<LoQ	7	<LoQ	34	60	306	5	21	3	<LoQ	1	3	<LoQ	1	6	29	<LoQ	2	<LoQ
		C	<LoQ	<LoQ	11	<LoQ	5	<LoQ	27	45	252	4	18	2	<LoQ	1	2	<LoQ	1	5	17	<LoQ	1	<LoQ
	T2	A	<LoQ	<LoQ	3	<LoQ	1	<LoQ	15	23	86	2	8	1	<LoQ	<LoQ	<LoQ	<LoQ	<LoQ	1	4	<LoQ	<LoQ	<LoQ
		B	<LoQ	<LoQ	5	<LoQ	7	1	27	60	173	7	19	3	<LoQ	1	2	<LoQ	2	2	3	<LoQ	2	<LoQ
		C	<LoQ	<LoQ	5	<LoQ	5	<LoQ	21	45	179	6	16	2	<LoQ	1	2	<LoQ	1	2	4	<LoQ	1	<LoQ
	T3	A	<LoQ	<LoQ	5	<LoQ	19	4	35	65	117	16	14	4	<LoQ	3	2	3	<LoQ	2	3	<LoQ	3	<LoQ
		B	<LoQ	<LoQ	11	2	109	26	60	117	238	50	27	11	1	8	5	11	2	4	6	<LoQ	9	<LoQ
		C	<LoQ	<LoQ	11	2	90	19	43	80	204	32	20	8	1	9	6	12	<LoQ	4	5	<LoQ	7	<LoQ
	T4	A	<LoQ	<LoQ	4	<LoQ	17	3	24	29	78	9	8	2	<LoQ	1	1	2	<LoQ	2	4	<LoQ	1	<LoQ
		B	<LoQ	<LoQ	10	2	110	16	59	77	155	31	26	7	2	6	5	9	3	4	8	<LoQ	3	<LoQ
		C	<LoQ	<LoQ	9	1	76	12	38	49	113	22	19	5	1	5	4	8	1	4	6	<LoQ	2	<LoQ
	T5	A	<LoQ	<LoQ	6	<LoQ	22	3	27	26	67	8	9	2	2	4	4	4	1	1	5	<LoQ	<LoQ	<LoQ
		B	<LoQ	<LoQ	15	2	106	13	54	55	140	24	23	5	4	7	8	10	2	2	9	<LoQ	1	<LoQ
		C	<LoQ	1	18	2	97	12	62	56	136	21	21	5	6	11	12	14	4	3	9	<LoQ	1	<LoQ

All values (µg/kg) are means of three replicates (*n* = 3) and expressed as syringol gentiobioside equivalents. Gu = guaiacol; Cr = cresol; Ph = phenol; Sy = syringol; MGu = 4-methylguaiacol; MSy = 4-methylsyringol; MG = monoglucosides; GG = gentiobiosides; PG = pentosylglucosides; RG = rutinoside. LoQ = limit of quantitation (1 µg/kg).

Table 3. Correlation matrix of phenolic glycoside concentrations in ng per berry from grapes of Chardonnay (a) and Shiraz (b).

	MGuGG	SyGG	MSyGG	PhPG	CrPG	GuPG	MGuPG	SyPG	MSyPG	PhRG	CrRG	GuRG	MGuRG	PhMG	CrMG	GuMG	SyMG
**GuGG**	0.941	0.924	0.864	0.910	0.817	0.958	0.853	0.939	0.894	0.938	0.896	0.963	0.898	0.439	0.201	0.353	0.115
**MGuGG**		0.938	0.962	0.768	0.862	0.923	0.913	0.895	0.972	0.805	0.933	0.939	0.963	0.411	0.230	0.459	0.241
**SyGG**			0.935	0.777	0.731	0.880	0.818	0.969	0.906	0.822	0.853	0.915	0.919	0.463	0.228	0.401	0.119
**MSyGG**				0.634	0.780	0.823	0.877	0.854	0.964	0.689	0.906	0.869	0.965	0.350	0.228	0.472	0.288
**PhPG**					0.728	0.914	0.698	0.861	0.711	0.969	0.715	0.865	0.703	0.552	0.187	0.280	0.017
**CrPG**						0.899	0.921	0.694	0.874	0.686	0.862	0.838	0.840	0.548	0.388	0.578	0.365
**GuPG**							0.909	0.901	0.906	0.905	0.899	0.970	0.894	0.550	0.329	0.477	0.152
**MGuPG**								0.765	0.963	0.710	0.967	0.921	0.951	0.463	0.466	0.656	0.381
**SyPG**									0.852	0.908	0.813	0.922	0.866	0.468	0.194	0.326	0.054
**MSyPG**										0.746	0.964	0.933	0.989	0.423	0.330	0.570	0.311
**PhRG**											0.771	0.906	0.760	0.457	0.154	0.771	0.022
**CrRG**												0.953	0.978	0.385	0.344	0.547	0.328
**GuRG**													0.945	0.482	0.317	0.483	0.162
**MGuRG**														0.404	0.320	0.555	0.297
**PhMG**															0.438	0.429	0.028
**CrMG**																0.652	0.292
**GuMG**																	0.378

(a)

	MGuGG	SyGG	MSyGG	PhPG	CrPG	GuPG	MGuPG	SyPG	MSyPG	PhRG	CrRG	GuRG	MGuRG	PhMG	CrMG	GuMG	SyMG
**GuGG**	0.925	0.822	0.564	0.875	0.476	0.629	0.529	0.795	0.486	0.920	0.920	0.971	0.927	0.891	0.474	0.474	0.053
**MGuGG**		0.909	0.788	0.901	0.666	0.787	0.751	0.871	0.717	0.771	0.969	0.873	0.989	0.778	0.602	0.323	0.217
**SyGG**			0.872	0.862	0.683	0.775	0.818	0.933	0.779	0.589	0.823	0.709	0.905	0.717	0.702	0.303	0.208
**MSyGG**				0.698	0.809	0.825	0.961	0.810	0.950	0.327	0.687	0.459	0.769	0.439	0.728	0.130	0.525
**PhPG**					0.749	0.795	0.746	0.936	0.709	0.743	0.883	0.808	0.909	0.867	0.727	0.323	0.181
**CrPG**						0.890	0.927	0.808	0.934	0.293	0.604	0.392	0.647	0.447	0.749	0.068	0.622
**GuPG**							0.875	0.856	0.872	0.428	0.711	0.542	0.752	0.530	0.695	0.219	0.480
**MGuPG**								0.836	0.993	0.307	0.656	0.424	0.729	0.441	0.783	0.101	0.602
**SyPG**									0.807	0.586	0.803	0.689	0.871	0.768	0.773	0.259	0.235
**MSyPG**										0.268	0.627	0.387	0.698	0.401	0.787	0.076	0.648
**PhRG**											0.832	0.971	0.784	0.887	0.285	0.461	0.004
**CrRG**												0.916	0.983	0.824	0.572	0.326	0.170
**GuRG**													0.887	0.897	0.380	0.439	0.030
**MGuRG**														0.818	0.643	0.328	0.187
**PhMG**															0.475	0.368	0.017
**CrMG**																0.193	0.323
**GuMG**																	0.015

(b)

Values are means of three replicates (n = 3) per variety from all sampling points and blocks measured as syringol gentiobioside equivalents. Gu = guaiacol; Cr = cresol; Ph = phenol; Sy = syringol; MGu = 4-methylguaiacol; MSy = 4-methylsyringol; MG = monoglucosides; GG = gentiobiosides; PG = pentosylglucosides; RG = rutinoside.
